# Eosinophilic Endomyocardial Fibrosis and Strongyloides stercoralis: A Case Report

**DOI:** 10.5812/cardiovascmed.9370

**Published:** 2013-05-20

**Authors:** Zahra Alizadeh-Sani, Anoushiravan Vakili-Zarch, Majid Kiavar, Behdad Bahadorian, Abas Nabavi

**Affiliations:** 1 Rajaei Cardiovascular Medical and Research Center, Tehran University of Medical Sciences, Tehran, IR Iran

**Keywords:** Endomyocardial Fibrosis, Strongyloides stercoralis, Magnetic Resonance Imaging

## Abstract

A 64-year-old female with history of previous aortoiliac occlusion and aortoiliac bypass operation four months ago presented with dyspnea, ascites and leg edema. She has been suffering from bloody diarrhea since two weeks earlier. Laboratory data showed important eosinophilia and stool examination was positive for Strongyloides stercoralis. Patient had clinical signs of heart failure. A cardiac MRI revealed hypersignal subendocardium in favor of endomyocardial fibrosis. Hypereosinophilic syndrome is defined by persistent hypereosinophilia for more than 6 months. The association with different etiologies is known but the report of cardiac involvement due to S. stercoralis infection is not very common. Cardiac manifestation is characterized by a restrictive cardiomyopathy due to toxic damage produced by activated eosinophils.

## 1. Case Report

**Figure 1.**
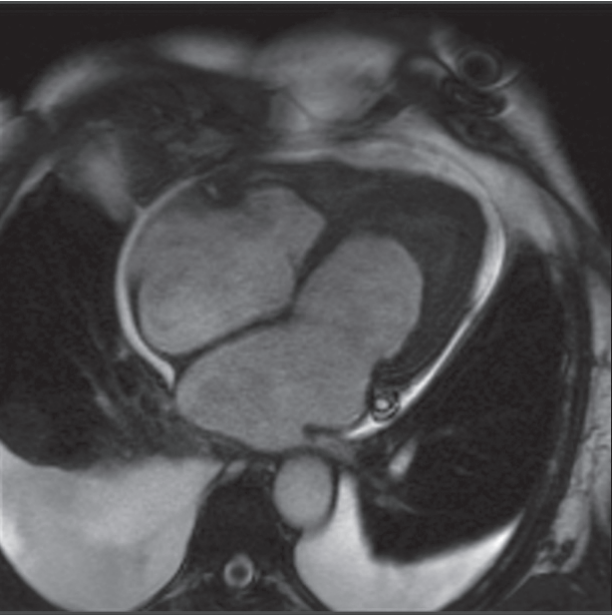
MRI Four Chamber View Through Both Ventricles Showed Apical Filling of Both Ventricles by Thrombus. Significant Pleural Effusion is Also Noted

**Figure 2.**
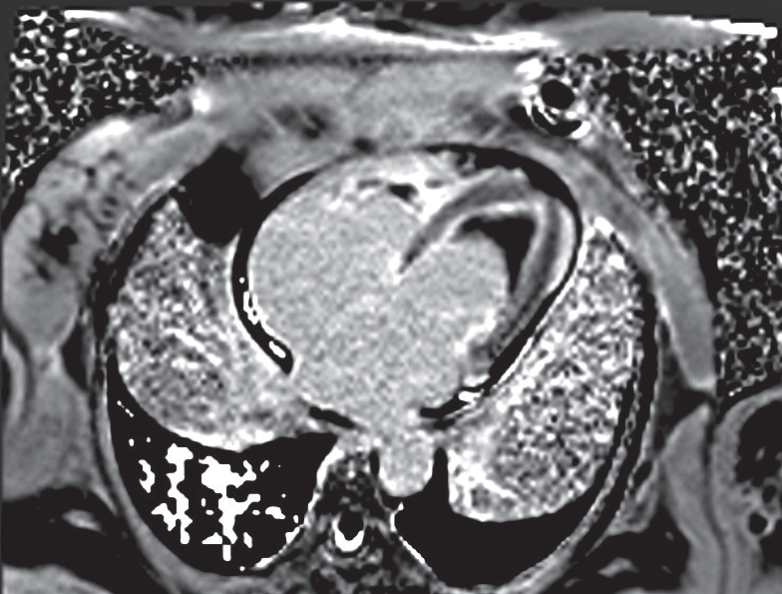
Delayed Enhanced Sequence in the Same View 10 MinutesAfter Gd-DTPA Infusion

A 64-year-old female with history of previous aortoiliac occlusion and aortoiliac bypass operation four months ago presented with dyspnea, ascites and leg edema. She has been suffering from bloody diarrhea since two weeks earlier. Laboratory data showed important eosinophilia in count of 2100 cells/µL and stool examination was positive for S. stercoralis. Patient had clinical signs of heart failure and standard treatment of heart failure started. Chest radiography revealed an enlarged cardiac shadow with a congestive vascular pattern The ECG showed sinus tachycardia with normal QRS morphology and duration. The transthoracic echocardiogram revealed left ventricular ejection fraction of 35%, severe diastolic dysfunction, moderate pulmonary hypertension and severe mitral and tricuspid regurgitation with apical filling of the both ventricles. A cardiac MRI revealed normal wall thickening in pre- and postgadolinium diethylenetriamine pentaacetic acid (Gd-DTPA) infusion dynamic sequences (*Figure 1*) with a large thrombus filling the apex. A delayed-enhancement sequence 10 minutes after infusion of 0.2 mmol Gd-DTPA per kilogram of body weight emphasized a hypersignal in the subendocardium (*Figure 2*). Thrombus is characterization by no evidence of early or late enhancement and of myocardial fibrosis by late gadoilnium enhancement (LGE). The delayed hyperenhancement explained by myocardial scarring which increases gadolinium concentration. The diagnosis of Endomyocardial fibrosis (EMF) was made on the basis of this typical MRI finding. Cardiac catheterization and biopsy was scheduled but unfortunately was not performed due to patient’s preference. Patient received Ivermectin for S. stercoralis treatment and medication for heart failure. On 6 month follow up she still has dyspnea with minimal exertion. She did not accept the risk of surgery.

## 2. Discussion

Hypereosinophilic syndrome is defined by persistent hypereosinophilia for more than 6 months. The association with different etiologies is known but cardiac involvement due to *S.* *stercoralis* infection is not that common (1). Cardiac manifestation is characterized by a restrictive cardiomyopathy due to toxic damage produced by activated eosinophils (2). It provokes endomyocardium fibrosis with obliteration of the right and left ventricles (3). Cardiac MRI may represent an important tool for early diagnosis and management. The presence of specific pattern of LGE may be alternative for cardiac biopsy in diagnosis of EMF (4). The LGE pattern commonly observed in EMF is the presence of fibrotic tissue only in the subendocardium and continuously extending from the subvalvular region to the apex of the ventricles. Surgery to remove the fibrotic tissue is the recommended in patients with NYHA functional classes III and IV.
